# Stereoisomers of the Bacterial Volatile Compound 2,3-Butanediol Differently Elicit Systemic Defense Responses of Pepper against Multiple Viruses in the Field

**DOI:** 10.3389/fpls.2018.00090

**Published:** 2018-02-22

**Authors:** Hyun G. Kong, Teak S. Shin, Tae H. Kim, Choong-Min Ryu

**Affiliations:** ^1^Molecular Phytobacteriology Laboratory, Infectious Disease Research Center, Korea Research Institute of Bioscience and Biotechnology, Daejeon, South Korea; ^2^Crop Protection R&D Center, Farm Hannong Co., Ltd., Nonsan-si, South Korea

**Keywords:** bacterial volatile compound, 2, 3-butanediol, plant viral disease, ISR, pepper

## Abstract

The volatile compound 2,3-butanediol, which is produced by certain strains of root-associated bacteria, consists of three stereoisomers, namely, two enantiomers (2R,3R- and 2S,3S-butanediol) and one meso compound (2R,3S-butanediol). The ability of 2,3-butanediol to induce plant resistance against pathogenic fungi and bacteria has been investigated; however, little is known about its effects on induced resistance against viruses in plants. To investigate the effects of 2,3-butanediol on plant systemic defense against viruses, we evaluated the disease control capacity of each of its three stereoisomers in pepper. Specifically, we investigated the optimal concentration of 2,3-butanediol to use for disease control against *Cucumber mosaic virus* and *Tobacco mosaic virus* in the greenhouse and examined the effects of drench application of these compounds in the field. In the field trial, treatment with 2R,3R-butanediol and 2R,3S-butanediol significantly reduced the incidence of naturally occurring viruses compared with 2S,3S-butanediol and control treatments. In addition, 2R,3R-butanediol treatment induced the expression of plant defense marker genes in the salicylic acid, jasmonic acid, and ethylene signaling pathways to levels similar to those of the benzothiadiazole-treated positive control. This study reports the first field trial showing that specific stereoisomers of 2,3-butanediol trigger plant immunity against multiple viruses.

## Introduction

A wide range of plant pathogens cause diseases in crops, leading to serious losses in yields, increased labor and costs, and human health problems caused by the use of harmful chemicals (Agrios, 2005; Strange and Scott, 2005; Lamberth et al., 2013). Among plant diseases, the incidence of viral diseases has been increasing yearly due to global warming, which is causing serious problems because there are currently no practical control methods for these diseases (Mandadi and Scholthof, 2013; Guerret et al., 2016; Jones, 2017). To date, the control of viral disease has primarily involved monitoring and chemical management of vectors that mediate disease progression, as well as virus resistance-based breeding and transgenic-based gene technology (Perring et al., 1999; Kang et al., 2005; Hashimoto et al., 2016; Khalid et al., 2017). However, the use of these technologies is limited due to environmental safety issues and the large amount of time it takes to apply them to the field (Agrios, 2005; Valkonen, 2015).

Many studies have focused on the use of induced resistance, i.e., systemic acquired resistance (SAR) and induced systemic resistance (ISR), against a variety of viruses as an alternate method for plant virus control. In particular, SAR against viral diseases in plants can be induced by weakened viruses, and plants can be protected from various viral diseases through systemic or localized viral infection (Ross, 1961; Ryals et al., 1994). However, the use of viruses to induce SAR is limited by treatment difficulties and the low success rate of inducible resistance in plants. As an alternative, microbes (pathogenic and beneficial bacteria) and chemicals have been used to induce plant resistance. However, it is difficult to apply pathogens in order to elicit plant resistance in the field, and some chemicals have significant negative effects on vegetative and generative growth (Ross, 1961; Tally et al., 1999; Kloepper et al., 2004a). Therefore, an increasing number of studies have focused on the induction of resistance in crops without perturbing their growth using plant growth-promoting rhizobacteria (PGPR) (Raupach et al., 1996; Zehnder et al., 2000; Zehnder et al., 2001; Murphy et al., 2003; Ryu et al., 2004). The application of *Pseudomonas fluorescens* CHAO, *Pseudomonas putida* 89B61, and *Serratia marcescens* 90-166 reduced the development of symptoms caused by *Tobacco necrosis virus* or *Cucumber mosaic virus* (CMV) in cucumber and tomato via eliciting ISR (Maurhofer et al., 1994; Raupach et al., 1996; Zehnder et al., 2000). Spraying of *Bacillus amyloliquefaciens* strain 5B6 (isolated from a cherry tree leaf) was recently reported to delay the development of symptoms caused by CMV in pepper (Lee and Ryu, 2016). To obtain PGPR-mediated ISR in the field, the activity and population instability of the treated bacteria are important issues. To overcome the disadvantages of microbial instability in the field, scientists have attempted to identify bacterial determinants, such as lipopolysaccharides, siderophores, and bacterial metabolites, with similar effects *in vitro* and in greenhouse experiments (Leeman et al., 1995; Duijff et al., 1997; Ongena et al., 2005; Ryu et al., 2013).

*B. amyloliquefaciens* strain IN937a was recently shown to stimulate systemic resistance due to the release of bacterial volatile compounds (BVCs) (Kloepper et al., 2004b; Ryu et al., 2004; Choi et al., 2014). Treatment with BVCs from PGPR reduced symptom development and CMV accumulation in pepper seedlings (Song et al., 2013; Choi et al., 2014). BVCs are important chemicals in plant virus control because they can affect plant resistance and can be applied on a wide range of scales (Kesselmeier and Staudt, 1999; Ryu et al., 2004; Kai et al., 2009).

One such BVC, 2,3-butanediol, is produced by various bacteria such as *Bacillus* spp., *Aerobacter* spp., and *Klebsiella* spp. (Barrett et al., 1983; Voloch et al., 1985; Ryu et al., 2004; Kopke et al., 2011). This compound is used in the manufacture of synthetic industrial products, aviation fuel, explosives, plasticizers, and pharmaceuticals (Gupta et al., 2005). Treatment with 2,3-butanediol has beneficial effects on plants, such as growth promotion and the induction of systemic resistance in *Arabidopsis thaliana* and tobacco (Ryu et al., 2003, 2004; Han et al., 2006). Both 2,3-butanediol and 2-butanol affect plant physiology, promote growth, and induce defense against the insect pest *Spodoptera littoralis* in maize (*Zea mays*) (D’Alessandro et al., 2014). However, little is known about the efficacy and applicability of other 2,3-butanediol isomers to plants to control plant viruses.

In this study, we evaluated the efficacy of bacterial volatiles for managing common, economically important viral diseases of pepper in South Korea under greenhouse and field conditions. Since the effect of 2,3-butanediol on induced resistance against viruses has not yet been studied, we investigated its effect on multiple viral diseases. First, we determined the optimal concentration of 2,3-butanediol to use to control CMV and *Tobacco mosaic virus* (TMV) under greenhouse conditions. In addition, we examined the effect of 2,3-butanediol isomers on induced resistance against TMV. We then examined the use of two enantiomers (2R,3R and 2S,3S-butanediol) and one meso compound (2R,3S-butanediol) in the field to control viral diseases, which were alleviated by these treatments compared with the control. We measured virus levels in plants using five representative pepper viruses in South Korea, including CMV, TMV, *Pepper mottle virus* (PepMoV), *Tomato yellow leaf curl virus* (TYLCV), and *Tomato spotted wilt virus* (TSWV), by qRT-PCR using specific primers. We confirmed the induction of plant defense systems by examining the expression of molecular markers of induced resistance, including genes related to/encoding *Capsicum annuum pathogenesis-related 4* (*CaPR4*), *Ca chitinase 2* (*CaChi2*), *Ca phenylalanine-I ammonia-lyase* (*CaPAL*), Ca*SAR8.2*, *Ca 1-aminocyclopropane-1-carboxylic acid oxidase* (*CaACC*), and *Ca proteinase inhibitor 2* (*CaPIN2*). Treatment with 2R,3R- and 2R,3S-butanediol reduced the severity of diseases caused by naturally occurring plant viruses and increased the yield of mature pepper fruits. Collectively, 2,3-butanediol treatments had different effects on inducing systemic resistance and viral suppression, depending on the isomer. This is the first report on the use of different isomers of BVCs to induce resistance to viral pathogens in pepper.

## Materials and Methods

### Greenhouse Trials

The induction of resistance against CMV and TMV by 2,3-butanediol isomers (Sigma–Aldrich Corp., Korea; Lot number 237639 (2R,3R-), 300349 (2S,3S-), 361461 (2R,3S-) was investigated in the greenhouse. Pepper (*Capsicum annum* L. cv. Bukwang) seeds were surface-sterilized with 6% sodium hypochlorite, washed four times with sterile distilled water, and then maintained on Murashige and Skoog agar medium (Duchefa, Haarlem, Netherlands) at 25°C for 3 days until germination. Germinated seeds were then transplanted into soil-less medium (Punong Horticulture Nursery Media LOW, Punong Co. Ltd., Gyeongju, Korea). Seedlings were grown in controlled conditions at 25 ± 2°C under fluorescent light with an intensity of approximately 7,000 lux and a 12 h/12 h day/night cycle, and were then transferred to the KRIBB greenhouse facility in Daejeon, South Korea.

2,3-Butanediol was prepared at concentrations of 1, 5, and 10 mM. Four-week-old pepper seedlings were drenched with 20 mL of each diluted solution of 2,3-butanediol. Alternatively, seedlings were treated with 1 mM benzothiadiazole (BTH; Syngenta, Durham, NC, United States) and water as positive and negative controls, respectively.

CMV and TMV were maintained in *Nicotiana tabacum* by mechanical passage in a temperature-controlled greenhouse. The virus inoculum used throughout the experiments consisted of systemically infected *N. tabacum* tissue ground in 50 mM potassium phosphate buffer, pH 7.0, containing 10 mM sodium sulfite (1 g of tissue: 10 ml of buffer). All tissues were chilled prior to use and maintained on ice during inoculation.

The primary leaves were rub-inoculated with CMV or TMV 7 days after treatment with 2,3-butanediol, BTH, or water (control). ‘Mock’ inoculated plants were rub-inoculated with 50 mM potassium phosphate buffer. The experiment was repeated three times with 10 replications per experiment. Virus quantification was performed via qRT-PCR as described below at 2 weeks after virus inoculation.

### Field Trial

The field trial was conducted in Geumsan-gun, Chungcheongnam-do, South Korea (36°35′ 32.27′′North, 127°30′ 34.75′′East), where plants are affected by multiple viral diseases each year, in mid-April of 2016 and 2017. For the field study, all necessary permits were obtained from the owners of private lands. Pepper seedlings (*Capsicum annum* L. cv. Bukwang) were transplanted at a distance of 30 cm. Before transplanting, the furrows were covered with black polyethylene plastic film to prevent weed problems. To test induced resistance under field conditions, 1-month-old seedlings were drenched with 100 ml per plant of 1 mM 2,3-butanediol isomers (2R,3R form, 2S,3S form, and 2R,3S form) (Sigma–Aldrich Corp., Korea) and 1 mM BTH solution three times per month. The same volume of sterilized water was used as a negative control. Each treatment was replicated five times in a completely randomized block design and consisted of 20 plants per treatment.

### Measurement of Pepper Fruit Yields

To investigate whether 2,3-butanediol isomers and BTH influence pepper fruit yields under field conditions compared with the water control, yields were measured 100 days post-treatment (dpt). Red-colored fruits (only) were harvested twice from mid-August to the end of August, depending on the growing season. Total yield (g/plant) per treatment was estimated, and the total fruit weight per plant was calculated for each harvest time. In addition, the number of fruits per plant was recorded for each harvest, and the total harvest was then calculated as the number of fruits/plant.

### Diagnosis of Viral Diseases

To evaluate plant virus levels in the field, 30 leaves per replication were randomly sampled 90 days after the application of 2,3-butanediol isomers and BTH. The leaves were immediately frozen in liquid nitrogen for total RNA extraction. Total RNA was isolated from the leaves using Tri reagent (Molecular Research Inc., Cincinnati, OH, United States) according to the manufacturer’s instructions and as described in our previous study (Lee et al., 2017). First-strand cDNA synthesis was performed with 2 μg of DNase-treated total RNA, oligo-dT primers, and Moloney murine leukemia virus reverse transcriptase (Enzynomics, Daejeon, Korea). The qRT-PCR assays consisted of cDNA, iQ^TM^ SYBR^®^ Green Supermix (Bio-Rad Inc., Hercules, CA, United States), and 10 pM of each primer. The cycling parameters were as follows: initial polymerase activation for 10 min at 95°C; followed by 40 cycles of 30 s at 95°C, 60 s at 55°C, and 30 s at 72°C. The identification of plant viruses as CMV, TMV, PepMoV, TYLCV, or TSWV was performed using the specific primer pairs shown in **Table 1**. *CaUBQ* (ubiquitin) was used as a loading control to ensure that equal amounts of RNA were used in each assay. Relative transcript levels were normalized with respect to *CaUBQ* mRNA levels and calculated using the 2^-ΔΔC_T_^ method. Standard error of mean values among replicates were calculated using Bio-Rad manager (version 2.1) (Bio-Rad CFX Connect).

**Table 1:** Primer sets used for the amplification of plant viruses and expression analysis of defense-related genes in this study.

	Primer	Sequence (5′-3′)
Plant	CMV CP Forward	5′-CGTTGCCGCTATCTCTGCTAT-3′
pathogenic	CMV CP Reverse	5′-GGATGCTGCATACTGACAAACC-3′
virus	TMV Forward	5′-ATGTCTTACAGTATCACTAC-3′
	TMV Reverse	5′-TCAAGTTGCAGGACCAGAGG-3′
	PepMoV Forward	5′-CAAGCAAGGGTATGCATGT-3′
	PepMoV Reverse	5′-AAGATCAGACACATGGA-3′
	TYLCV Forward	5′-CGCCCGCCTCGAAGGTTC-3′
	TYLCV Reverse	5′-TCGTCGCTTGTTTGTGCCTTG-3′
	TSWV Forward	5′-ATGTCTAAGGTTAAGCTCAC-3′
	TSWV Reverse	5′-TCAAGCAAGTTCTGCGAGTT-3′

Plant	*CaUBQ Forward*	5′-GCACAAGCACAAGAAGGTTAAG-3′
defense-related	*CaUBQ Reverse*	5′-GCACCACACTCAGCATTAGGA-3′
gene	*CaPR1 Forward*	5′-ACTTGCAATTATGATCCACC-3′
	*CaPR1 Reverse*	5′-ACTCCAGTTACTGCACCATT-3′
	*CaPR2 Forward*	5′-TTTTAGCTATGCTGGTAATCCGCG-3′
	*CaPR2 Reverse*	5′-AAACCATGAGGACCAACAAAAGCG-3′
	*CaPR4 Forward*	5′-AACTGGGATTGAGAACTGCCAGC-3′
	*CaPR4 Reverse*	5′-ATCCAAGGTACATATAGAGCTTCC-3′
	*CaPIN2 Forward*	5′-TGGGACTTTCATTTGTGAAGGAGAG-3′
	*CaPIN2 Reverse*	5′-GACACAGTGAATAGGCAATATTTGG-3′
	*CaPAL Forward*	5′-ATTCGCGCTGCAACTAAGAT-3′
	*CaPAL Reverse*	5′-CACCGTGTAAGGCCTTGTTT-3′
	*CaACC Forward*	5′-AGTGGCCTTCAACTCCTCAA-3′
	*CaACC Reverse*	5′-TTCCGTTTGTGATCACCTCA-3′
	*CaSAR8.2 Forward*	5′-TAGTGAGACTAAGAAAGTTGGACG-3′
	*CaSAR8.2 Reverse*	5′-AAGAGTGCATGCAGTATCACAAAG-3′
	*CaChi2 Forward*	5′-ATTGGACGATGGAAGCCATCACCAG-3′
	*CaChi2 Reverse*	5′-ATATTCCGAATGTCTAAAGTGGTAC-3′
	*CaActin Forward*	5′-CACTGAAGCACCCTTGAACCC-3′
	*CaActin Reverse*	5′-GAGACAACACCGCCTGAATAGC-3′

### Assessment of Defense-Priming Gene Expression

To analyze the expression of defense-priming genes, the expression of candidate priming genes was analyzed using the primers shown in **Table 1** (Yang et al., 2009, 2011; Song et al., 2013). The expression of defense-priming genes in pepper leaves was analyzed using the same leaves sample as the assay of viral disease. As a control to ensure that equal amounts of RNA were analyzed in each experiment, relative RNA levels were calibrated and normalized to the level of *CaActin* mRNA (GenBank accession no. AY572427).

### Statistical Analysis

The experimental data sets were subjected to analysis of variance using JMP software ver. 4.0 (SAS Institute Inc., Cary, NC, United States^1^). The significance of differences among treatments was determined based on the magnitude of the *F* value at *P* = 0.05. When a significant *F* value was obtained for the treatments, separation of means was accomplished using Fisher’s protected least-significant difference (LSD) at *P* = 0.05.

## Results

### Optimization of 2,3-Butanediol Levels to Elicit Induced Resistance under Greenhouse Conditions

**FIGURE 1:**
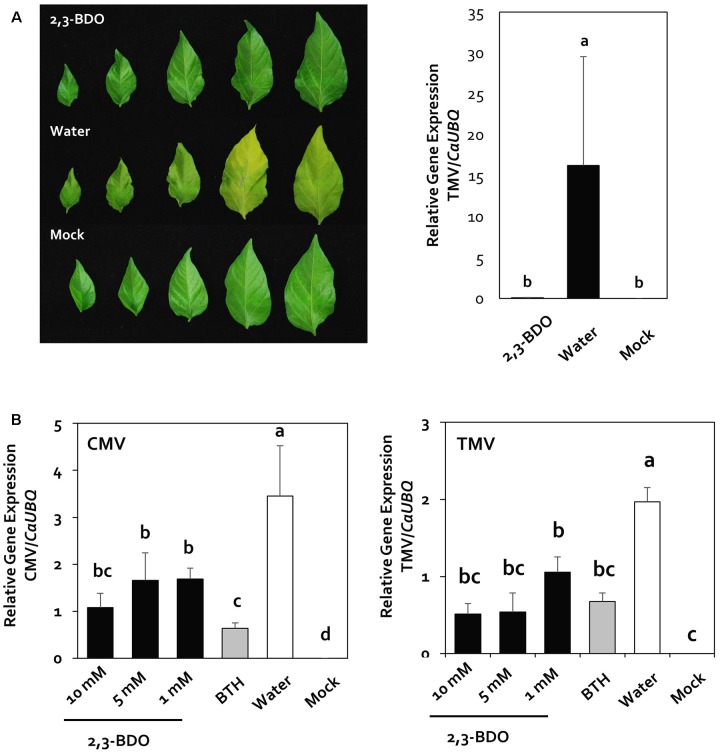
Disease suppression of rub-inoculated viruses by drench application of 2,3-butanediol (2,3-BDO). **(A)** Disease symptoms caused by rub-inoculated *Cucumber mosaic virus* (CMV) were evaluated 2 weeks post-inoculation, and the induction of resistance against *Tobacco mosaic virus* (TMV) by 2,3-BDO was validated in the greenhouse. **(B)** Validation of the induction of resistance against CMV and TMV by treatment with three concentrations of 2,3-BDO in the greenhouse. Expression of viral-specific genes was measured 2 weeks after treatment of pepper plants with 1, 5, and 10 mM 2,3-BDO, 1 mM benzothiadiazole (BTH), and water. Bars represent mean ± SEM (*n* = 10). The housekeeping gene *CaUBQ* was used as a reference in qPCR.

In our previous study (Choi et al., 2014), we found that the use of 1 mM 2,3-butanediol reduced the severity of bacterial spot caused by *Xanthomoas axonopodis* pv. *vesicatora* in pepper. However, it was not clear whether this compound induces resistance activity against plant viral pathogens. To evaluate whether 2,3-butanediol induces plant immunity against CMV and TMV under greenhouse conditions, we quantified the viruses 2 weeks after infection in the greenhouse. After TMV inoculation, we observed yellowing caused by the virus in the leaves of the control plants, whereas no symptoms were observed in the leaves of plants under 2,3-butanediol treatment (**Figure 1A**). In addition, the quantitative value of the virus (i.e., the relative gene expression level compared with *CaUBQ*) in the 2,3-butanediol treatment group was 0.1, which was dramatically less than that of the control, at 16.25 (**Figure 1B**). Furthermore, to determine the minimum concentration of 2,3-butanediol needed, we examined the effect of 10, 5, and 1 mM 2,3-butanediol on CMV and TMV. Based on the quantification of CMV, there was no statistically significant difference among the 10, 5, and 1 mM 2,3-butanediol treatments, with relative gene expression levels of 1.07, 1.67, and 1.69, respectively; these values were three times less than the control (3.45) under all treatments. CMV levels were lowest after BTH treatment, at 0.63 (**Figure 1B**). The relative gene expression levels of TMV were 0.52, 0.54, and 1.06 under 10, 5, and 1 mM 2,3-butanediol treatment, respectively. Therefore, the concentration of virus was highest under 1 mM 2,3-butanediol treatment, but there was no statistically significant difference among treatments. Under all treatments, CMV levels were significantly lower than those in the control. Under BTH treatment, the relative gene expression level of CMV was 0.68, which was not significantly different from that detected under 1 mM 2,3-butanediol treatment, the lowest concentration of this compound tested (**Figure 1B**). Therefore, the minimum effective concentration of 2,3-butanediol was 1 mM (*P* < 0.05, *n* = 10), which was used in subsequent experiments.

### Assessment of the Protective Effects of 2,3-Butanediol against Viral Diseases under Field Conditions

**FIGURE 2:**
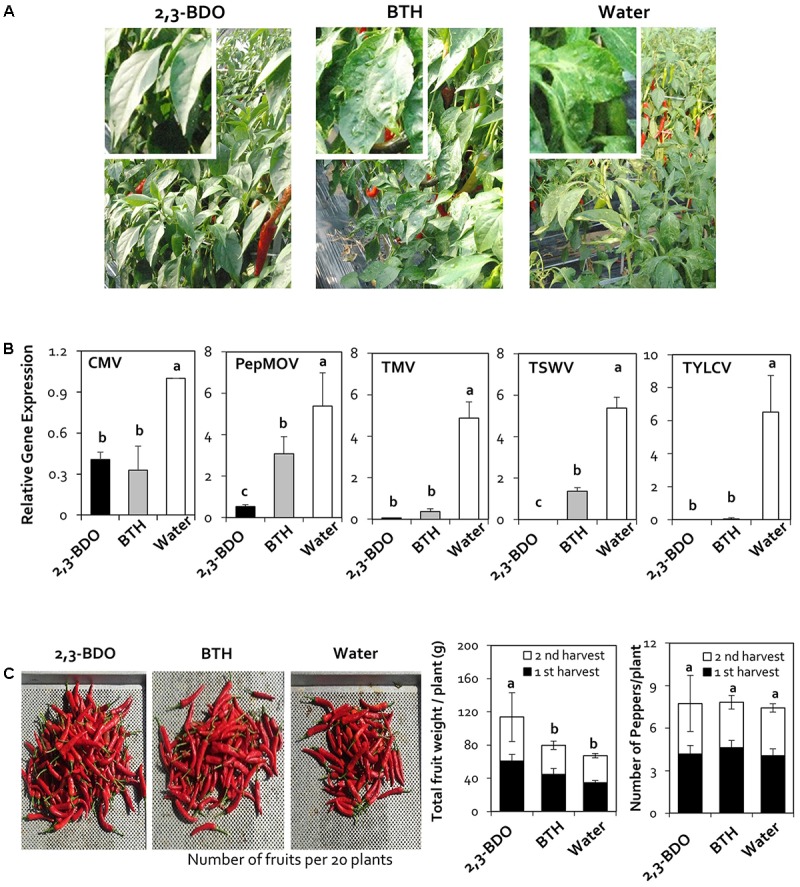
Disease suppression of naturally occurring viruses by treatment with 2,3-butanediol (2,3-BDO). **(A)** Disease symptoms caused by naturally occurring viruses were evaluated at 90 days post-transplantation (dpt). **(B)** The induction of resistance against *Cucumber mosaic virus* (CMV), *Pepper mottle virus* (PepMoV), *Tobacco mosaic virus* (TMV), *Tomato spotted wilt virus* (TSWV), and *Tomato yellow leaf curl virus* (TYLCV) by application of 2,3-BDO was evaluated at 90 dpt in the field. Bars represent mean ± SEM (*n* = 100). The housekeeping gene *CaUBQ* was used as a reference in qPCR. **(C)** Fruit fresh weight per plant and the fruit yield of 20 plants treated with 2,3-BDO, benzothiadiazole (BTH), and water were assessed at 100 dpt. Different letters indicate statistically significant differences (*P* = 0.05). Error bars represent mean ± SEM.

To evaluate virus resistance under field conditions in 2016, we treated field-grown pepper plants with 2,3-butanediol three times at 1 month intervals. We sampled leaves at 90 days after planting and performed quantitative analysis of five viruses: CMV, PepMoV, TMV, TSWV, and TYLCV. In the water-treated control, the five viruses (CMV, PepMoV, TMV, TSWV, and TYLCV) were detected at levels of 1.00, 5.38, 4.87, 5.38, and 6.53, respectively. Plants treated with 2,3-butanediol had viral levels of 0.41, 0.06, and 0.00 for CMV, TMV, and TYLCV, respectively, which did not significantly differ from the values obtained under BTH treatment (0.33, 0.36, and 0.06, respectively). Under 2,3-butanediol treatment, PepMoV and TSWV were detected at levels of 0.53 and 0.00, respectively, which were lower than the levels detected under BTH treatment (3.08 and 1.36, respectively). Therefore, 2,3-butanediol can be successfully used to control various viruses under field conditions (**Figures 2A,B**).

To investigate the effect of 2,3-butanediol on red pepper fruit yields as well as the ability of the treatment to control viruses, we examined fruit yield in 2017. For the yield measurements, only red pepper was harvested in the second round, and the weight and number of fruits were measured. In the field experiment, the number of fruits did not significantly differ between the treatments; however, fruit weight per plant was 113.7 g upon 2,3-butanediol treatment, an increase of 1.7-fold compared with the control value of 67.2 g (**Figure 2C**).

**FIGURE 3:**
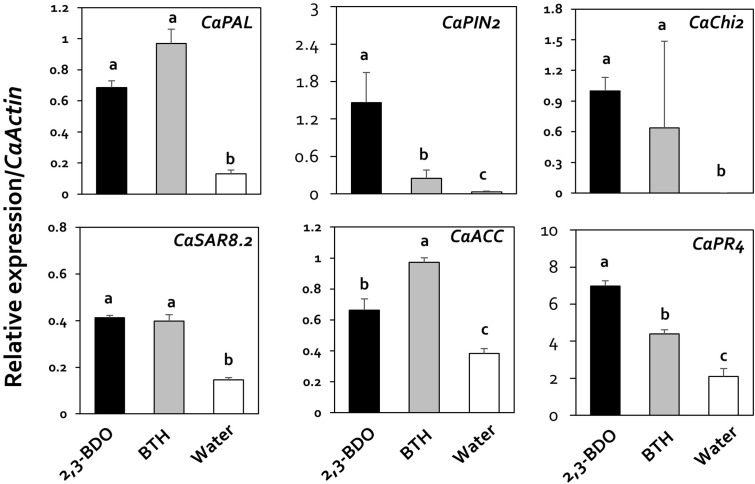
Induction of pepper defense-related gene expression by 2,3-butanediol under field conditions. Quantification of the expression of marker genes of the salicylic acid (*CaPAL*, *CaPR4*, and *CaSAR8.2*), ethylene (*CaACC*), and jasmonic acid (*CaPIN2*, *CaPR4*, and *CaChi2*) signaling pathways in plants treated with 1 mM 2,3-butanediol, 1 mM benzothiadiazole (BTH), and water. The housekeeping gene *CaActin* was used for normalization. Different letters within panels indicate significant differences between treatments according to LSD at a significance level of *P* < 0.05. Error bars represent mean ± SEM. Sample size: *n* = 100 plants per treatment in 2016.

Previous studies showed that viral suppression in plants is caused by the immune response (Mandadi and Scholthof, 2013). Therefore, in the current study, we investigated the mechanism underlying viral suppression in response to 2,3-butanediol treatment by examining the expression of genes related to jasmonic acid (JA), salicylic acid (SA), and ethylene (ET) biosynthesis and signaling, as these plant hormones play a central role in the regulation of plant immune responses. The specific genes in the JA, SA, and ET pathways that we examined were *CaPAL* (SA), *CaPIN2* (JA), *CaChi2* (JA), *CaSAR8.2* (SA), *CaACC* (ET), and *CaPR4* (SA and JA). The relative expression levels of these genes in response to 2,3-butanediol treatment (compared with *CaActin*) were 1.46, 6.98, 1.00, 0.69, 0.66, and 0.41, with increases of 46.4-, 3.3-, 10-, 5.2-, 1.7-, and 2.8-fold, respectively, compared with the control (**Figure 3**). In particular, *CaPIN2* and *CaPR4* were more highly upregulated by 2,3-butanediol than by BTH treatment (**Figure 3**). Taken together, these results indicate that 2,3-butanediol induces the expression of plant defense marker genes in pepper, which could be associated with the induction of three major defense hormone signaling pathways, the SA, ET, and JA pathways, in these plants.

### Effect of 2,3-Butanediol Isomers on Induced Resistance against Naturally Occurring Viral Diseases

**FIGURE 4:**
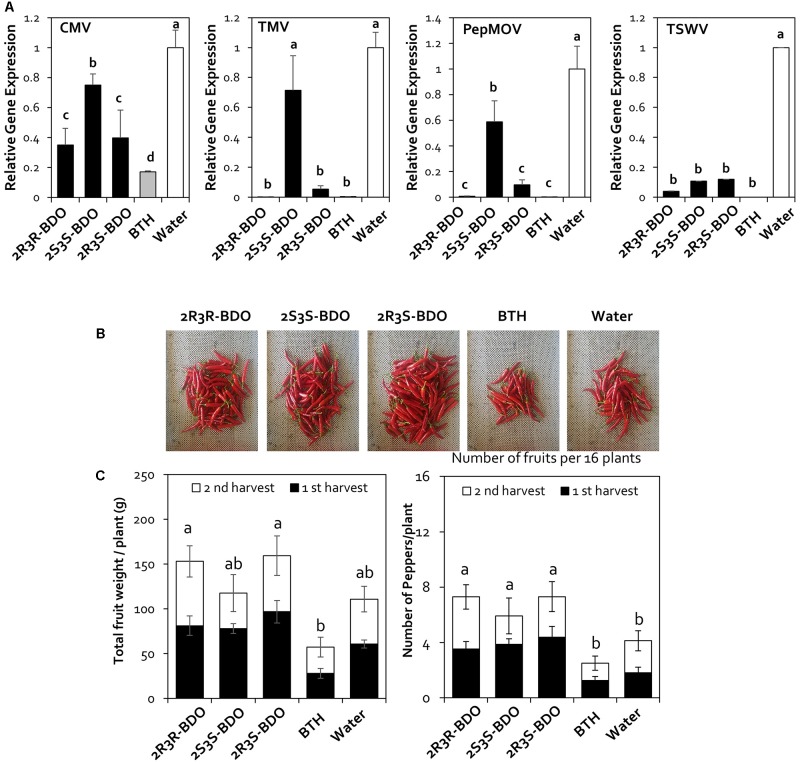
Disease suppression of naturally occurring viruses by treatment with 2,3-butanediol (2,3-BDO) isomers. **(A)** Disease symptoms caused by naturally occurring viruses were evaluated at 90 days post-transplantation (dpt). **(B)** The induction of resistance against *Cucumber mosaic virus* (CMV), *Tobacco mosaic virus* (TMV), *Pepper mottle virus* (PepMoV), and *Tomato spotted wilt virus* (TSWV) by application of each 2,3-BDO isomer (2R,3R-, 2R,3S-, and 2S,3S-butanediol) was evaluated at 90 dpt in the field. Bars represent mean ± SEM (*n* = 100). The housekeeping gene *CaUBQ* was used as a reference in qPCR. **(C)** Fruit fresh weight per plant and the fruit yield of 16 plants treated with 2,3-BDO, benzothiadiazole (BTH), and water were assessed at 100 dpt. Different letters indicate statistically significant differences (*P* < 0.05). Error bars represent mean ± SEM.

In the natural ecosystem, microorganisms can produce three 2,3-butanediol isomers, i.e., the R form (2R,3R-butanediol), S form (2S,3S-butanediol), and meso form (2R,3S-butanediol) depending on the species (Herold et al., 1995; Celińska and Grajek, 2009; Ji et al., 2011; Kandasamy et al., 2016). After the first year of greenhouse and field trials, we investigated whether the different isomers of 2,3-butanediol affect ISR. Treatment with 2,3-butanediol has an effect on virus control through increasing plant immunity. Therefore, we investigated the effects of the other isoforms of 2,3-butanediol in a second field experiment in 2016. We performed a quantitative assay of pepper viruses using the same five viruses as in the first field experiment but failed to detect TYLCV in the second field experiment. Treatment with 2R,3R-butanediol (R form) led to a 2.8-, 689.7-, 122.9-, and 25.9-fold decrease in CMV, TMV, PepMoV, and TSWV levels, respectively. Compared with 2R,3R-butanediol, the effect of 2R,3S-butanediol (meso form) treatment on these four viruses did not significantly differ from that of treatment with the other forms of 2,3-butanediol and BTH (**Figure 4A**). Plants treated with 2S,3S-butanediol (S form) showed a 9.2-fold decrease in TSWV levels compared with the control, which was not significantly different from the other treatments. However, under this treatment, the levels of CMV, TMV, and PepMoV were reduced by only 1.3-, 1.4-, and 1.7-fold, making 2S,3S-butanediol less effective than the other forms of 2,3-butanediol and BTH (**Figure 4A**). Therefore, the effects of the R form and meso form of 2,3-butanediol were similar to that of BTH treatment, whereas the S form of 2,3-butanediol had less of an effect than the other isomers.

**FIGURE 5:**
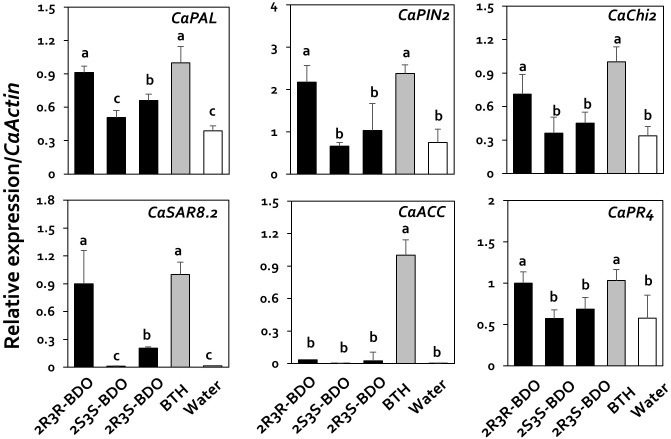
Induction of pepper defense-related gene expression by 2,3-butanediol isomers under field conditions. Quantification of the expression of marker genes of the salicylic acid (*CaPAL*, *CaPR4*, and *CaSAR8.2*), ethylene (*CaACC*), and jasmonic acid (*CaPIN2*, *CaPR4*, and *CaChi2*) signaling pathways in plants treated with 1 mM of each 2,3-butanediol isomer (2R,3R-, 2R,3S-, and 2S,3S-butanediol), 1 mM benzothiadiazole (BTH), and water. The housekeeping gene *CaActin* was used for normalization. Different letters within panels indicate significant differences between treatments according to LSD at a significance level of *P* < 0.05. Error bars represent mean ± SEM. Sample size: *n* = 100 plants per treatment in 2017.

To confirm the effects of these compounds, we investigated the expression of the plant immunity genes by qRT-PCR. Under 2R,3R-butanediol treatment, *CaPIN2*, *CaPR1*, *CaChi2*, *CaPAL*, *CaSAR8.2*, and *CaPR4* were upregulated 2.9-, 15.7-, 2.1-, 2.4-, 54.8-, and 1.7-fold, respectively, compared with water treatment (**Figure 5**). Under 2R,3R-butanediol treatment, the expression levels of these six genes were the highest among 2,3-butanediol isomer treatments, with no significant difference from BTH treatment (**Figure 5**). The expression levels of *CaPAL* and *CaSAR8.2* under 2R,3S-butanediol treatment were 0.7 and 0.2, respectively, i.e., 1.7- and 12.5-times that of the water-treated control. However, these levels were lower than those measured under 2R,3R-butanediol and BTH treatments (**Figure 5**). By contrast, in plants treated with 2S,3S-butanediol, the expression levels of these genes did not significantly differ from those detected in the water-treated control (**Figure 5**).

Therefore, the decrease in virus levels detected under 2R,3R-butanediol and BTH treatment was confirmed by the expression patterns of the plant defense genes.

### Measurement of Pepper Fruit Yields

To investigate the effects of 2,3-butanediol isomers on red pepper fruit yields as well as the ability of the treatment to control viruses, we examined fruit yield in 2017. For the yield measurements, only red pepper was harvested in the second round, and the weight and number of fruits were measured. When we measured the yield of red pepper fruit in response to isomer treatment in the field in 2017, the number of fruits per plant was 7, 6, and 7 per plant for plants treated with the R, S, and meso form of 2,3-butanediol, respectively, which was significantly higher than that of the BTH-treated group (3) and the control (4). The fruit weight was 116.8, 94.7, and 117 g in plants treated with the R, S, and meso forms of 2,3-butanediol, respectively, which was significantly higher than in plants treated with the BTH (40 g) and control (66 g) treatments (**Figure 4B**).

Based on the results of the 2 year field experiments, the R form of 2,3-butanediol is effective in controlling plant viral diseases and increasing the yields of red pepper fruit. These results are important for the practical application of volatile substances produced by microorganisms under field conditions. In addition, this is the first report that different 2,3-butanediol isomers have different effects on the control of plant viruses under field conditions.

## Discussion

Our study demonstrates that drench application of the BVC 2,3-butanediol to roots induces plant systemic defense responses in pepper resulting in the reduced accumulation of viruses in leaves. In addition, we found that treating plants with different isomeric compounds of 2,3-butanediol had different effects on inducing plant immunity, depending on the structure of the bacterial-derived volatile substance. This is the first study showing that 2,3-butanediol controls plant viral diseases and the plant’s immune response is dependent on the presence of specific 2,3-butanediol isomers. The results are based on the quantification of plant defense gene expression through qRT-PCR. Therefore, 2,3-butanediol isomers can potentially be used as a bioprotectant of plants against viruses under field conditions.

Pepper fruit is an important commercial vegetable product. Viral diseases have recently been reducing the yield and quality of red peppers yearly in South Korea. Under natural field conditions, various plant viruses infect pepper, such as CMV, TMV, TSWV, PepMoV, and TYLCV. TSWV, PepMoV, and TYLCV occur sporadically, whereas CMV and TMV are the most important red pepper viruses. CMV, PepMoV, and TMV belong to group IV (viruses with positive sense single-stranded RNA); the majority of plant viruses are included in this group. TSWV belongs to group V (viruses with negative sense single-stranded RNA), and TYLCV belongs to group II (viruses with single-stranded DNA). In field experiments, different viral diseases may occur depending on the abiotic conditions (topography, climate, and soil type). Plant viruses depend on insect vectors for their survival, transmission, and spread. Consequently, changes in insect vectors affect the occurrence of viral diseases.

The effectiveness of many bacteria in controlling viral diseases has been demonstrated (Ryu et al., 2004; Dashti et al., 2012). We recently evaluated the effects of leaf-colonizing *B. amyloliquefaciens* strain 5B6 in protecting crop plants against CMV in pepper (Lee and Ryu, 2016). Although bacterial isolates are effective in controlling viral diseases, their application in the field is limited due to the decreased survival and activity of introduced bacteria in the natural environment (Vejan et al., 2016). Therefore, the inhibition of plant viral diseases using active substances produced by bacteria has been proposed, and BVCs released from bacteria have been shown to promote growth and induce defense responses in the host plant (Bailly and Weisskopf, 2012; Yi et al., 2016). However, the effect of 2,3-butanediol on plant viral disease has not previously been elucidated. In this study, we investigated the effects of soil application of 2,3-butanediol on protecting plants from various viruses. We determined the optimal concentration of 2,3-butanediol for field application through analysis in the greenhouse and monitored the reduction in virus levels in the field. The qRT-PCR assay showed that viral accumulation continuously decreased in 2,3-butanediol-treated plants in both the greenhouse and field (**Figures 1**, **2**). The levels of TYLCV, TSWV, and PepMoV also decreased under 2,3-butanediol treatment, suggesting that 2,3-butanediol could be used as a commercial formulation for the biological control of viral diseases.

The direct inhibition of virus accumulation in plants by 2,3-butanediol treatment might be due to its effect on the induction of ISR in plants. Indeed, the inhibition of viral disease through induced resistance mediated by bacteria and bacterial metabolites in plants has been reported (Murphy et al., 2003). The effect of 2,3-butanediol treatment appears to be dependent on SA, JA, and ET for ISR induction under field conditions (**Figure 3**). These results suggest that the reduction in viral infection by 2,3-butanediol treatment is not due to its antiviral effect against viruses but is instead due to its direct effect on increasing plant resistance to overall viral diseases through the induction of plant immunity. Similarly, the direct effect of 2,3-butanediol on pepper roots was reflected by increases in plant defense gene expression. Indeed, *CaPAL*, *CaSAR8*.*2*, *CaACC*, and *CaPR2* were previously shown to be upregulated in plants whose roots were drenched with 2,3-butanediol (Yi et al., 2016). However, 2,3-butanediol has not previously been shown to protect plants from viral diseases, although treatment with the bacterial volatile derivative 3-pentanol was found to mitigate the severity of disease in pepper caused by *Xanthomonas axonopodis* and naturally occurring CMV. RT-PCR analysis showed that this treatment increased the expression of defense-related genes involved in the SA, JA, and ET signaling pathways (Choi et al., 2014). The suppression of plant viral diseases by both 2,3-butanediol and 3-pentanol treatment suggests that additional BVCs with this effect might also be discovered.

All three isomeric forms of 2,3-butanediol are reportedly produced by specific types of bacteria. Therefore, using field experiments, we confirmed that the plant defense response against plant viruses likely occurs in an isomer-dependent manner. Specifically, in plants treated with the R form and meso form of 2,3-butanediol, the accumulation of viruses continuously decreased, as was observed in the other field experiment using mixed isomers (**Figure 4**). However, in plants treated with the S form of this compound, there was no significant difference in viral levels compared with the control. The mechanism underlying the induction of plant resistance by specific 2,3-butanediol isomers is unclear. Furthermore, we examined the expression levels of *CaPR4*, *CaPIN2*, *CaPAL*, *CaACC*, *CaSAR8*.*2*, and *CaChi2* to directly determine the effects of the 2,3-butanediol isomers on inducing plant immunity. We found that plant defense-related genes were induced only by 2R,3R-butanediol (**Figure 5**). Indeed, the stereochemistry of 2R,3R-butanediol is important for ISR in tobacco to *Erwinia carotovora* subsp. *carotovora* SCC1, as 2S,3S-butanediol did not activate plant resistance to this pathogen (Han et al., 2006). These findings suggest that the induction levels of plant defense responses differ depending on the isomers of the BVCs utilized.

The isomer-dependent activity of 2,3-butanediol in plants could be due to three underlying mechanisms. First, the 2,3-butanediol isomers investigated in this study are enriched in bacteria that produce similar isomers in the rhizosphere. Indeed, 2,3-butanediol is produced by various bacteria including *B. amyloliquefaciens* (Celińska and Grajek, 2009), *Bacillus subtilis* (Xiao and Xu, 2007), *Enterobacter aerogenes* (Byun et al., 1994), *Klebsiella pneumoniae* (Biebl et al., 1998), *Klebsiella oxytoca* (Syu, 2001), *Lactococcus lactis* (Hugenholtz and Starrenburg, 1992), *Paenibacillus polymyxa* (De Mas et al., 1988), and *S. marcescens* (Zhang et al., 2010). Soil bacteria and fungi synthesize the three stereoisomers (2R,3R-, 2S,3S-, and 2R,3S-) of 2,3-butanediol from pyruvate via a dehydrogenation and oxidation step (Wang et al., 2014; Kandasamy et al., 2016; Yi et al., 2016). While the exact role played by 2,3-butanediol between the bacteria and plant host is largely unknown, the production of various 2,3-butanediol isomers, depending on the bacteria, suggests that they might be used for bacterial interactions. For example, 2,3-butanediol from *B. subtilis* negatively regulates *RsmA*, encoding a post-transcriptional regulator of virulence factors, in *P. carotovorum* subsp. *carotovorum* (Kõiv et al., 2013; Broberg et al., 2014). Therefore, 2R,3R- and 2R,3S-butanediol, which are mainly produced by *Bacillus* spp., are likely enriched in *Bacillus* spp. producing the same isomer in the rhizosphere. Specific strains of *B. amyloliquefaciens*, *B. subtilis*, *B. pasteurii*, *B. cereus*, *B. pumilus*, *B. mycoides*, and *B. sphaericus* elicit significant reductions in disease levels in various hosts (Kloepper et al., 2004b). The number of whitefly nymphs that transmit *Tomato mottle virus* (ToMoV) was significantly reduced by treatment with *B. amyloliquefaciens* IN937a and *B. subtilis* IN937b, and significant reductions in the severity of ToMoV and in disease incidence resulted from *B. subtilis* IN937b and *B. pumilus* SE34 treatment (Murphy et al., 2000).

Second, 2,3-butanediol has direct effects on the host plant, depending on the isomer used. Treatment with 2,3-butanediol directly affects plant immunity. Treatment with the 2,3-butanediol production mutant *B. subtilis* failed to stimulate plant defense responses, whereas treatment with wild-type *B. subtilis* successfully induced plant defense responses against bacterial pathogens, indicating that 2,3-butanediol plays a direct role in plant immunity (Ryu et al., 2004; Rudrappa et al., 2010). The different effects of the three 2,3-butanediol isomers on pepper indicate that plants have distinct receptors for each of these isomers. Consistent with our data, only 2R,3R-butanediol effectively improves plant growth and resistance to *P. carotovorum* subsp. *carotovorum* in *Nicotiana*, suggesting that plants have specific receptors for this isomer (Han et al., 2006). BVCs from *B. subtillis* GB03 do not promote the growth of *Arabidopsis thaliana* with mutated *cre1*, which encodes a cytokinin receptor (Ryu et al., 2003). This suggests that plant receptors play a role in the recognition of bacterial BVCs. BVCs may be recognized by ET receptors in plants. Indeed, it has been proposed that BVCs are recognized by ETR1-like ET receptors (Bailly and Weisskopf, 2012). However, we do not have any evidence that CRE1 can physically bind to bacterial volatiles such as 2,3-butanediol. Plants recognize bacteria by detecting microbe-associated molecular patterns via membrane-localized receptor kinases. Currently, receptor signaling is best understood at the genetic level. Indeed, genetic analyses demonstrated that ET receptor signaling negatively regulates ET responses (Hua et al., 1998). Specifically, this is achieved via receptor activation of CONSTITUTIVE RESPONSE1 (CTR1) protein kinase, which represses ET signaling mediated by ETHYLENE INSENSITIVE2 (EIN2). This mechanism was demonstrated by the findings that mutants with null mutations in multiple ET receptor genes and *ctr1* loss-of-function mutations display similar ET responses, whereas those with gain-of-function receptor mutations are insensitive to ET (Hua et al., 1998). Isomer-specific receptors in plants should be investigated based on the results of this and previous plant receptor studies.

Third, 2,3-butanediol might reduce the incidence of viral diseases in plants by providing an alkaline environment that protects bacterial cells from unfavorable acidic conditions (Yoon and Mekalanos, 2006; Pradhan et al., 2010; Bari et al., 2011). In addition, this treatment increases bacterial robustness against harmful compounds, such as root exudates. Furthermore, root exudates from 2,3-butanediol-treated pepper exhibited selective antagonism against *Ralstonia solanacearum.* By contrast, application of 2,3-butanediol-elicited root exudate increases robustness of the PGPR *Bacillus subtilis* GB03 and the saprophyte *Pseudomonas protegens* Pf-5 (Yi et al., 2016). These findings suggest that 2,3-butanediol increases the robustness of the acidic rhizosphere environment (Hinsinger et al., 2003; Huang and Chen, 2003). The increased robustness of the bacteria may be dependent on the activities of particular 2,3-butanediol isomers, which have different effects on the induction of ISR in plants. Based on these three possible mechanisms, the effects of 2,3-butanediol on plant immune responses can be explained not only by its direct effect on rhizosphere bacteria, but also by the direct induction of plant resistance and the changes in rhizosphere conditions such as plant root exudate.

## Conclusion

This is the first report demonstrating that 2,3-butanediol protects pepper plants from viral diseases under field conditions. In particular, through field experiments, we confirmed that different isomers of 2,3-butanediol have different effects on controlling plant viruses via the induction of plant immunity. Active compounds derived from bacteria (such as 2,3-butanediol) could be highly valuable for industrial applications. Despite the efficacy of 2,3-butanediol in protecting plants from viral diseases, the limitation of formulation technology to control volatility currently poses a challenge for the commercialization of 2,3-butanediol. However, further experiments are needed to assess the mechanism underlying the induction of ISR and the effects of the rhizosphere environment on a particular 2,3-butanediol isomer.

## Author Contributions

C-MR designed the study. HK, TS, and TK performed all the experiments. TS and TK helped with the *in vitro* experiments and *in vivo* treatments. C-MR contributed to scientific discussions that guided the project. C-MR and HK wrote the paper.

## Conflict of Interest Statement

TS and TK are employed by Farm Hannong Co., Ltd. (South Korea). The other authors declare that the research was conducted in the absence of any commercial or financial relationships that could be construed as a potential conflict of interest.

## Footnotes

1 www.sas.com
